# The identification of functional motifs in temporal gene expression analysis

**Published:** 2007-02-27

**Authors:** Jiuzhou Song, Jaime Bjarnason, Michael G. Surette

**Affiliations:** 1 Department of Animal and Avian Sciences, and University of Maryland, Maryland 20742, USA; 2 Department of Microbiology and Infectious Diseases, and Department of Biochemistry and Molecular Biology, Health Sciences Centre, University of Calgary, Calgary, AB, Canada, T2N 4N1

**Keywords:** Gene expression, motif, FDR, mixed models

## Abstract

The identification of transcription factor binding sites is essential to the understanding of the regulation of gene expression and the reconstruction of genetic regulatory networks. The *in silico* identification of cis-regulatory motifs is challenging due to sequence variability and lack of sufficient data to generate consensus motifs that are of quantitative or even qualitative predictive value. To determine functional motifs in gene expression, we propose a strategy to adopt false discovery rate (FDR) and estimate motif effects to evaluate combinatorial analysis of motif candidates and temporal gene expression data. The method decreases the number of predicted motifs, which can then be confirmed by genetic analysis. To assess the method we used simulated motif/expression data to evaluate parameters. We applied this approach to experimental data for a group of iron responsive genes in *Salmonella typhimurium* 14028S. The method identified known and potentially new ferric-uptake regulator (Fur) binding sites. In addition, we identified uncharacterized functional motif candidates that correlated with specific patterns of expression. A SAS code for the simulation and analysis gene expression data is available from the first author upon request.

## Introduction

Gene expression exhibits temporal and spatial patterns in response to environmental changes and as part of developmental and differentiation processes. The binding of transcription factors (TFs) to regulatory elements of genes controls when and where specific genes will be expressed. The rate of gene transcription is regulated largely by the TFs that bind and affect the affinity of RNA polymerase for the transcription initiation site of the gene. The identification and testing of relevant TF binding sites remains a significant challenge in functional genomics (Tompa *et al*. 2005).

Traditionally, TF binding sites have been characterized by experimental methods. The availability of complete genome sequences enables us to use computational tools and advanced statistical methods to predict new potential TF binding sites. In addition, recent advances in high throughput gene expression analysis technologies can provide large amounts of detailed expression data. These techniques include DNA microarray(Conway and Schoolnik 2003; Eisen *et al*. 1998; Spellman *et al*. 1998), SAGE (serial analysis of gene expression) (Angelastro *et al*. 2000) and *in vivo* gene expression using promoter reporters(Anderson *et al*. 1988; Bjarnason *et al*. 2003; Blouin K. 1996; Kalir *et al*. 2001; Setty *et al*. 2003; Van Dyk *et al*. 2001; Zaslaver *et al*. 2004) and *in vivo* TF binding techniques(Beer and Tavazoie 2004; Braas *et al*. 2003; Elemento and Tavazoie 2005; Pritsker *et al*. 2004; Rosenfeld *et al*. 2005) (Ui *et al*. 1998).

Thorough the comparison of expression profiles, genes or putative genes can be grouped based on similarity of expression profiles by cluster analysis. Within the same cluster, genes are assumed to be transcriptionally co-regulated, and upstream regions of these co-expressed genes can be searched for shared sequence motifs. High conservation of upstream sequence motifs has lead to the widespread use of multiple alignments to search for conserved upstream nucleotide sequences (Conlon *et al*. 2003; Eskin and Pevzner 2002; J. van Helden 2000; J. van Helden 1998; Sinha and Tompa 2000) for motif discovery in several eubacterial species (McGuire *et al*. 2000) and *Sacchromyces. cerevisiae* (Frederick P. Roth 1998; Hughes *et al*. 2000). Although the strategies can identify many significant repeats or conserved sequences upstream of the coding region, the statistically significant meaning of the putative motifs is based solely on the frequencies of the nucleotides or patterns against the genome species. It doesn’t indicate the probability that the putative motifs are TF binding sites or have biological relevance for gene expression (Caselle *et al*. 2002; Cora *et al*. 2004), and these putative TF sites must be confirmed by wet-bench genetic analysis. Compared with relatively simple bacterial genomes, the TF binding sites in eukaryotes tend to be much shorter and the size of the potential regulatory region much larger, consequently the number of the predicted putative motifs will be greater. Confirming all putative motifs in all organisms by wet bench experimental analysis becomes challenging. Therefore approaches that would decrease the number of putative sites and efficiently obtain functional motifs are crucial issues in the *in silico* analysis of regulatory sites. Using the combined analysis of complete genome information with gene expression data it is possible to identify statistically significant putative motifs. However, the current motif discovery methods enable us to overestimate the putative motifs compared to what we expect to be significant from biological data (Cora *et al*. 2004; Cora *et al*. 2005). Using traditional statistical methods, the identification and testing of functional motifs involves multiple comparison tests, and the avoidance of Type I error, where a null hypothesis is incorrectly rejected, can be problematic. Although some researchers have tried to explore analysis techniques to address these issues (Keles *et al*. 2002; Kessler and Witholt 2001), the present status of research suggests that the exploration and application of the new analysis techniques would be advantageous

In this paper, we adopted the method of controlling the false discovery rate (FDR) (Benjamini 1995) to decrease type I error and estimated motif candidate effects with longitudinal model (Wolfinger *et al*. 2001). We are interested in identifying putative functional motifs within co-regulated genes derived from temporal expression data. In the current study, we demonstrate that controlling the FDR and motif effect estimation are more appropriate for functional motif detection, and illustrate the strategy via a simulation study and time series gene expression data in *Salmonella typhimurium.*

## Materials and Methods

### Definition of false of the false discovery rate (FDR)

The FDR is the expected proportion of true null hypotheses erroneously rejected out of the total number of null hypotheses rejected (Benjamini 1995). In theory, if R null hypotheses are rejected in multiple comparison tests, V is the number of true null hypotheses erroneously rejected. FDR is defined as:

FDR=E(V|R|R>0)P(R>0)

Assume that m, the number of multiple comparison tests, are simultaneously tested, there are m null hypotheses *H*_1_, *H*_2_, …, *H_m_* on basis of independent test statistics *Y*_1_, *Y*_2_ …, *Y_m_*, from each *Y_i_*, figuring out corresponding p-values, *P*_1_, *P*_2_ …, *P_m_*, then denoting the ordered values as *P*_(1)_ ≤ P_(2)_ ≤ … ≤ *P*_(_*_m_*_)_, *P*_(1)_, being the most significant and *P*_(_*_n_*_)_ the least significant in the usual terminology. The values to control FDR when *P*_(_*_i_*_)_ are independently distributed are given by the step-up formula:

$$k=\text{max}\{I:P_{\left(i\right)}\leq (I|m)q\}.$$

We reject *P*_(1)_, *P*_(2)_, …, *P*_(__k__*)_; if no such k exists, we reject none. It has been proven that the FDR could be controlled at some level, *q* (Benjamini 1995). That is, out of k hypotheses rejected, it is expected that the proportion of erroneously rejected hypotheses is not greater than the FDR adjusted p-value.

### Analysis of simulated data

**Figure 1:**
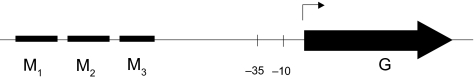
The simulated gene structure. *M*_1_, *M*_2_ and *M*_3_ are three simulated transcriptional factor binding sites and the basal promoter element represented by the -35 and -10 regions. The combination of the three motifs with the basal promoter element was random. The motif effects could be negative, positive or have no effect.

The simulated data was generated by Monte Carlo simulation. We simulated 10 promoters that were associated with 50 sequence motifs: 8 functional motifs (two motifs with negative effect and six motifs with positive effects) and 42 nonfunctional motifs. The simulation was run 50 times and the simulated gene structure is shown in Figure 1. We assume that each gene has a conserved expression profile, three motifs upstream of the gene, and that motif effects are additive. A positive effect indicates that the TF site would work to enhance or activate gene expression, and a negative effect indicates that the TF site works to repress or hinder gene expression.

Here we temporarily ignore non-linear interaction among motifs and assume that the effects of multiple motifs are additive. All combinations between promoters and motifs have random uniform distribution. The simulated parameters are shown in Table 2; the simulated model is as follows:

$$Y_{i}=G_{i}+\sum_{j}\sum_{k}Motif_{jk}+\sum_{j}\sum_{k}Moti{f_{jk}}^{*}Motif_{jk}+{ɛ}_{i}$$

Here, *Y_i_* is gene *i* expression level; *G_i_* is the ith gene conserved expression profile, *i* = 1,2, …, 10; *Motif_jk_* is the *k*th motifs additive effects in the *j*th cluster; *j*,*k* are the number of cluster and motifs, respectively. ɛ*i* is the *i*th normal random effects.

To check family-wise error rate (FWER), we shuffled the motif order against gene expression level 50 times to obtain the permutated data. For the simulated motifs, we tested by *t*-test for each of 50 motifs in both the simulated data and the permutated data. Under the assumption of unequal variances, the approximate *sig* statistic is computed as

$$sig=\frac{\left({\bar{x}}_{i}-\bar{x}\right)}{\sqrt{w_{1}+w_{2}}}$$

where $w_{1}=\frac{s_{1}^{2}}{n_{1}},w_{2}=\frac{s_{2}^{2}}{n_{2}}$,

$$df=\frac{{\left(w_{1}+w_{2}\right)}^{2}}{\left[w_{1}^{2}/(n_{1}-1)]+[w_{2}^{2}/(n_{2}-1)\right]}$$

sig is the significant value of statistics; *x̄_i_* is the mean of the ith candidate motif in a cluster and given gene expression experiment; *x̄* is the mean of a cluster and given gene expression experiment; *n*_1_ is the number of the *i*th candidate motif in a given cluster and gene expression experiment; *n*_2_ is the total number of a given cluster and gene expression experiment.

After the 50 tests were ordered by *P*_(_*_i_*_)_, the FWER and the FDR were determined as described above.

### Analysis of real gene expression data and estimation of motif effect in *S. typhmurimum*

A previous study by our group (Bjarnason *et al.* 2003) identified iron responsive genes in *S. typhimurium* by screening a random promoter library in hogh and low iron. Expression profiles for the iron response clones were further organized on the basis of their expression profile across 11 conditions and 5–8 time points using cluster analysis (Eisen *et al.* 1998). Cluster analysis arranges genes according to their similarity in patterns of gene expression. Genes previously demonstrated to be repressed by the transcriptional regulator Fur were found within one of the larger clusters. Fur is primary transcriptional regulator involved in the regulation of iron uptake and metabolism.

We took 300 base pairs (bp) of upstream sequences of each gene in this cluster and tried to find sequence patterns from the unaligned DNA sequences. We adopted the Mismatch Tree Algorithm (MITRA) and MEME – approaches to obtain composite regulatory patterns that are groups of monad patterns that occur near each other (Bailey 1999; Eskin and Pevzner 2002). The MITRA found 58 dyad motifs of length 6bp or greater in this set of co-regulated genes. We used unequal variance *t*-test where a significant *t*-value is indicative of a putative motif or composite pattern affecting the gene expression in the condition of that time point. The FDR adjusted p-value was computed as described above.

In the screened motif candidates we obtained consensus candidates. In order to quantitatively evaluate the motif candidates, we estimate the motif candidates with a longitudinal model. Let the random variable *Y_ij_* = *Y_t_*(*t_ij_*) denote the gene expression level of *ithgene,* measured at *t_ij_* in each experiment. We then assume that *Y_ij_* satisfies

$$Y_{ij}=\beta_{1j}+\beta_{2i}t_{ij}+\beta_{3i}t_{ij}^{2}+{ɛ}_{ij},j=1,\ldots ,n_{i}$$

Where *n_i_* is the number of longitudinal measurements available for the *i*th gene, and where all error components *ɛ_ij_* are assumed to be independently normally distribution with mean zero and variance *σ*^2^. The *Y_ij_* can be rewritten as

$$Y_{i}=Z_{i}\beta_{i}+{ɛ}_{i}$$

Where *Y_i_* equals (*Y_i_*_1_, *Y_i_*_2_, …, *Y_ini_*)′, *ɛ_i_* equals (*ɛ_i_*_1_, *ɛ_i_*_2_ …, *ɛ_ini_*)′, *βi* equals (*β*_1_*_i_*, *β*_2_*_i_*, *β*_3_*_i_*)′, and *Z_i_* i*s* the (*n_i_* × 3) matrix, the columns of which contain only ones, all time points *t_ij_* and all squared time points *t*^2^*_ij_*. The above model can now be seen as a linear regression model, and the vector *β_i_* of unknown parameters can be estimated by replacing *Y_i_* in the ordinary least squares estimator *β_i,OLS_* = (*Z*′*_i_Z_i_*)^−1^ *Z*′*_i_Y_i_*, by the vector of observed value, leading to *β*.

All analysis processes were implemented by SAS.

## Results and discussion

### Simulated data

**Figure 2:**
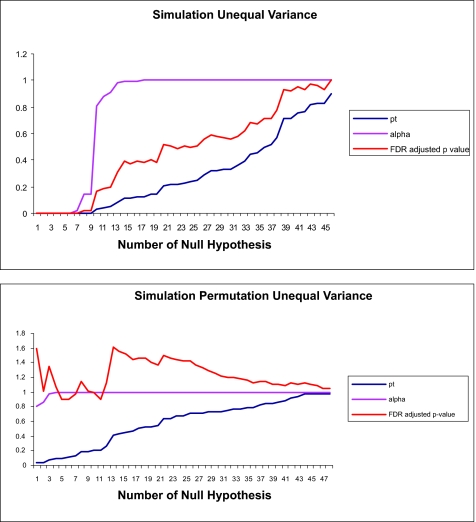
A. The plot of FDR adjusted p-value, experiment-wise type I error (FWER)(alpha) and comparison-wise type I error (CWER)(pt) in the simulated data set with ten genes and eight functional motifs and 50 motif-gene expression combinations. The *x-axis* is the number of hypotheses rejected and the *y-axis* is he probability level for the different statistical tests. B. The plot of FDR adjusted p-value, experimentwise type I error (FWER) (alpha) and com-parisonwise type I error (CWER)(pt) in the shuffled simulation data set with ten genes and eight functional motifs and 50 motif-gene expression combinations. The *x-axis* is the number of hypotheses rejected and the *y-axis* is he probability level for the different statistical tests.

In order to evaluate the different statistical analysis methods, a simulated data set was generated by combining regulatory motifs with basic promoter elements, as illustrated in Figure 1. The false discovery rate (FDR) adjusted p-value, familywise (or experimentwise) error rate (FWER) and comparison-wise error rate (CWER) computed from the *t*-probabilities in the simulated data set with ten genes and eight functional motifs are plotted in Figure 2A. The first 13 comparisons in the simulated data and first seven comparisons in permutated data are shown in Table 3. From Figure 2A, at very low probabilities of null hypotheses, FDR adjusted p-value, CWER and FWER are very close. With increasing numbers of rejected hypotheses, the FDR adjusted *p-*value is always lower than the FWER and higher than the CWER. From Table 3, at *i* = 12, FDR adjusted *p-*value = 0.2016, FWER = 0.91096, CWER=0.04837, based on *t*-probability or CWER, 12 motifs are detected, which could be considered “true” functional motifs. Based on FWER<0.5 criteria, the FDR adjusted *p-*value =0.01778, nine functional motifs would be detected, all of the eight true motif in simulated data are in the detected motif list, at *i* = 8, FWER=0.1478, FDR adjusted p-value = 0.0200, CWER=0.0032. Thus, FDR adjusted p-value controlled FDR, the FDR is similar to the family wise rate, so in such a situation controlling the FDR adjusted p-value is same as the controlling of FWER. When the number of null hypotheses is less than that of all hypotheses under testing, the FDR adjusted *p-*value is much smaller than that of FWER.

### Permutation data

**Figure 3:**
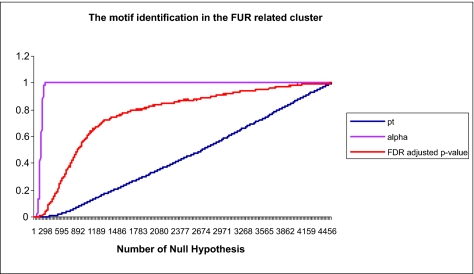
The plot of FDR adjusted p-value, experiment-wise type I error (FWER)(alpha) and comparison-wise type I error (CWER)(pt) in the fur-related cluster in a time series gene expression experiment in *S.typhimurium* with 3886 motif-gene expression combinations. The *x-axis* is the number of hypotheses rejected and the *y-axis* is he probability level for the different statistical tests.

In order to generate a negative data set, the putative motif and condition-time point associations calculated above were randomly permutated. The FDR adjusted p-value, FWER and CWER were determined and plotted in Figure 2B and also shown in Table 3. Because the relationships among the putative motifs and gene-condition-time points have been randomized, no null hypotheses should theoretically be rejected. As we can see in Figure 3, when the association between the putative motifs and expression data was shuffled, the FWER sharply increased. From Table 2, at *i* = 2, FDR adjusted p-value = 1.00346, FWER = 0.8656, CWER = 0.04014. Based on the CWER criteria, two motifs were tentatively detected which could be considered “true” functional motifs. However from FWER < 0.5 and the FDR adjusted p-value, nothing of significance was detected. The FDR adjusted p-value larger than one would imply that the number of Type I errors exceed the number of rejected hypothesis. These results illustrate how unreliable the CWER is in multiple comparison tests of motif discovery.

### Expression data from an iron-regulated cluster from *S. typhimurium*

We have previously characterized iron responsive genes in *S. typhimurium* (Bjarnason *et al*. 2003). Iron responsive genes were clustered on the basis of their expression profiles across 11 conditions and time points via cluster analysis- (Eisen *et al*. 1998), and one significant cluster containing known Fur responsive genes was selected for analysis. Fur mediates the majority of transcriptional repression to iron in bacteria (Earhart 1996). We adopted the Mismatch Tree Algorithm (MITRA) (Eskin and Pevzner 2002) to search for composite regulatory patterns in the 300bp sequence upstream of each gene. The MITRA found 58 dyad putative motifs of length 6bp or greater and the unequal variance *t-*values and their corresponding probabilities were calculated from the time series gene expression experiment. For the 3886 (67 time points by 58 dyad putative motifs) pattern-condition-time point association tests of the genes in the iron regulated cluster, the FDR, CWER and FWER are plotted in Figure 3. The behaviors of the indices are similar to those in Figure 2. At very low probabilities of null hypotheses, FDR adjusted p-value, FWER and CWER are very small and similar. For analysis of this real data, we take the FWER < 0.5, in this case, *i* = 63, FDR adjusted p-value = 0.0088, FWER= 0.4260 and CWER= 0.0001, that is only 63 null hypotheses out of 3886 association tests would be rejected. Adopting these criteria we would accept 22 significant DNA patterns out of 58 predicted MITRA DNA patterns. If extending criteria to the FDR adjusted p-value = 0.05, then *i* = 132, FDR adjusted p-value = 0.04894, FWER= 0.9984, CWER= 0.0017, then 132 null hypotheses would be rejected and 39 DNA patterns out of 58 putative DNA patterns would be accepted. We examined all of the 22 and 39 patterns from the two criteria, respectively, and using WebLogo (Crooks GE 2002) they could be grouped into three subgroup motifs based on overlapping sequence patterns. The averages of FDR adjusted p-value, CWER, and FWER values for the motif candidates are shown in Table 4, the minimum of FDR adjusted p-value is 0.0043. It is worth noting that the motif A candidate in the Table 4 is similar to reported Fur motif binding sequences (Earhart 1996).

**Figure 4:**
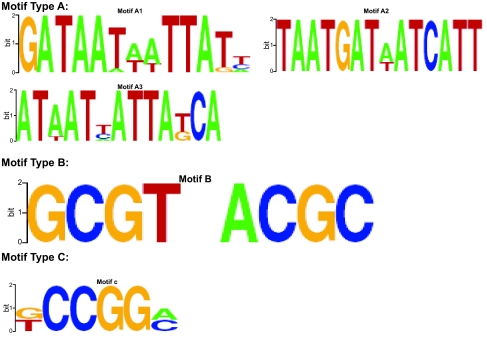
The LOGO graphical representation (Schneider and Stephens 1990) of predicted motif candidates in the iron regulated gene cluster. The images were generated using WebLogo (Crooks GE 2002) using the overlapping aligned patterns from the MITRA analysis and DFDR prediction.. The relative height of the base reflects the degree of conservation.

Graphical representations of the consensus sequences derived from WebLogo (Crooks GE 2002; Schneider and Stephens 1990) are shown in are Figure 4. Each logo consists of stacks of wDNA symbols for each position. The overall height of the stack indicates the sequence conservation (nucleotide presence/conservation) at that position, while the height of symbols within the stack indicates the relative frequency of each nucleic acid at that position. The sequence logo provides a visual description of a binding site. The predicted consensus for Motif A matches that of the published Fur consensus site (Earhart 1996). In addition to the known Fur binding sites in this set of promoters, additional Fur sites are predicted. Motif candidate B and C did not match any known transcription factor binding sites and may represent a new TF binding sites. This potential regulatory motif is currently being investigated experimentally.

### The estimation of the motif candidates via longitudinal model

In order to quantitatively describe the motif candidates, we estimated motif effects with a longitudinal model. Motif effects are defined as the motif candidates take effects for their locating genes over time, Table 5 shows motif effects which contain hypothesis tests for the significance of each of the motif and interaction effects which contain hypothesis tests for the interaction between time and motif, and indicates that fixed effects of the motif candidates and the interactions among motif candidates, time and quadratic time are very significant. Subsequently, the maximum likelihood (ML) and restricted maximum likelihood (REML) and minimum variance quadratic unbiased estimation (MIVQUE0) are used to estimating for all parameters in the longitudinal model. From Table 6, the estimations of the three methods for the parameters are the same, but the standard errors of the ML estimates are less than that of the REML and MIVQUE0, and the estimates and standard error of the REML are the same as that of MIVQUE0.

Further investigation of the estimates for the parameters shows that significant effects seem to be present among the motif candidates A and B, although they have opposite effects. The motif candidate C has the weakest effects (0.0438). There are significant positive interactions between motif candidate B and time effects, and weaker interactions between motifs A and C and time effects. The results also indicate that the interaction of all motif candidates and quadratic time effects are negative and weak, and suggest that motif influences gene expression level over time.

### Diagrams of functional motifs and predicted promoters

**Figure 5:**
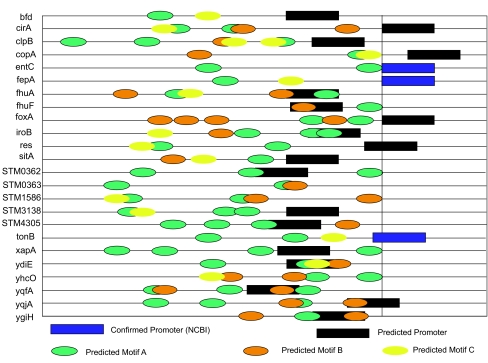
The distribution of motifs in the iron regulated genes cluster. The ORF is open reading frame starting point. The annotated or predicted promoter is indicated in blue and black, respectively. The positions of predicted motifs are indicated by the colored ovals.

To illustrate the distribution of the predicted motif candidates and the relationship between the functional motifs and promoters, we used BCM Search Launcher (Smith *et al*. 1996) to predict the position of the promoters. The positions of the potential motifs were mapped upstream of coding region. A motif occurrence is defined as a position in the sequence with a match that has a significant *p-*value and significant effects for gene expression levels (FDR<0.05). The ordering and spacing of all non-overlapping functional motif occurrences and the highest score promoters are shown for each upstream sequence in Figure 5. We find the distribution of the motifs is neither normal nor uniform. We also find that most of the genes are predicted to be regulated by more than one TF binding site, consistent with the control of transcription by comprehensive interactions among the DNA binding sites.

## Discussion

The past few years have witnessed a dramatic increase in our knowledge of primary genetic information at the level of genome sequences, which has been complemented by the development of methodologies for genome scale analysis of gene expression. The merging of these two knowledge bases provides an opportunity for rapid *in silico* analysis of genetic regulation. In principle there are many potential TF binding sites that can exist for any given gene. One of the fundamental challenges is the accurate prediction of TF binding sites and ultimately the estimation and evaluation of their qualitative and quantitative effects on gene expression. In addition to the specific TF binding site, contextual information can influence the quantitative effects of a particular site. This information includes surrounding DNA sequence effects (influencing such processes as DNA flexibility and intrinsic curvature), spacing with respect to promoter elements and combinatorial effects of multiple TF elements. These influences are not readily predicted from our current understanding of gene regulation and experimental verification is still required for many predicted TF sites. By combining gene expression data with motif prediction and the application of statistical analysis, the number of predicted TF binding sites can be significantly reduced with a greater degree of confidence (Cora *et al*. 2004; Cora *et al*. 2005) Here we have demonstrated that using an adjusted False Discovery Rate (FDR) and estimation of motif effects as a statistical strategy improve the prediction of real relative to false TF binding sites.

The combined analysis of motif prediction and gene expression data is complex, involving thousands of multiple comparison tests. Avoidance of type I error and efficiently identifying functional TF binding sites is not only of theoretical importance but will also reduce the amount of experimental work required for verification. The traditional approach to dealing with multiple comparisons is through the control of family-wise error rate (FWER), rather than controlling the “comparison-wise error rate” (CWER). FWER is the probability of one or more false rejections of true hypotheses, regardless of how many hypotheses are true and what value the parameters of the false hypotheses take. FWER is controlled by strictly setting the specific rejection threshold, so that the probability that any of the null hypotheses tested are erroneously rejected is below a specified low level. The false discovery rate (FDR), the expected ratio of erroneous rejections to the number of rejected hypotheses, gives us an alternative choice. In our simulation experiment, as documented by other researches (Dudoit 2003; Reiner *et al*. 2003; Storey and Tibshirani 2003a; Storey and Tibshirani 2003b), the FDR adjusted p-value is very similar to FWER when the number of null hypotheses is tested. In such a situation, controlling the FDR adjusted p-value is similar to controlling the FWER. Multiple comparison procedures controlling the FDR adjusted p-value are more powerful than the commonly used multiple comparison procedures based on FWER and CWER. FDR is well suited to large multiple comparison problems in which existing procedures lack power, especially for the preliminary identification and tests of functional motifs in large scale gene expression data and bundles of putative motifs.

The identification of putative regulatory motifs is another challenge in this research. The methods for discovering DNA patterns are directly related to the quality of putative motifs and the accuracy of building genetic networks. DNA pattern discovery methods (Alvis Brazma 1998; Eisen *et al*. 1998; Eskin and Pevzner 2002; J. van Helden 2000; J. van Helden 1998; Szymon M. Kielbasa 2001; Tompa *et al*. 2005; Zhou Zhu 2002), look at the significant patterns (J. van Helden 1998), monad or spaced dyads (Eskin and Pevzner 2002; J. van Helden 2000; Lars M. Jakt 2001) over the whole genome and are based on nucleotide frequencies and sampling probabilities; each one with its own advantages and disadvantages. Applying pattern discovery to a cluster of genes based on the similarity of their gene expression profiles is more advantageous than the strategy of using the entire genome (Eskin and Pevzner 2002; Hao Li 2002) and upstream DNA sequence multiple alignments (Frederick P. Roth 1998; Hertz and Stormo 1999; Sinha and Tompa 2002). Expression profile clustering associates genes controlled by a regulatory cascade even if it may involve many different TFs and binding sites (Harmen J. Bussemaker 2001).

To estimate motif effects, we used a longitudinal model. Longitudinal data means when the same measurement is made repeatedly on experimental units over time, inducing correlation in the measurements within an experimental unit. As compared with cross-sectional data analysis, modeling of longitudinal data presents additional difficulties in that we must specify the time trend of the population mean and the correlation structure of the observations, and how covariates affect both of these. The linear mixed models are extensions of linear regression models for longitudinal data. It contains fixed and random effects where the random effects are used to model between-subject variation and the correlation induced by this variation; it is an extremely flexible analysis tool. The estimation of motif effects by longitudinal model analysis that we present provides a method to obtain functional motifs from large scale of gene expression data sets. The gene expression longitudinal data is characterized by repeated observations over time on the same set of genes, and the main feature is that the repeated observations on the same gene tend to be correlated; the longitudinal model gives us an method to overcome the issue.

Identification of TF binding sites remains problematic. Combining gene expression data with motif searching techniques provides improved identification of regulatory sites. In the strategy presented here, the adjusted FDR and estimation of motif effects are demonstrated to provide a balance between false positive and false negative predictions. In the future, we will adopt this technique for genomic expression patterns (Lee *et al*. 2004; McCarroll *et al*. 2004) and control the proportion of false positive (Fernando *et al*. 2004) to improve the accuracy of functional motifs, these are likely to help us in functional footprinting of the regulatory motif, and the building of genetic networks.

## Figures and Tables

**Table 1:** Outcomes when testing m hypotheses

	*H*_0_ NOT rejected	*H*_0_ rejected	Total
*H*_0_ True	U	V	*m*_0_
*H*_0_ False	T	S	m-*m*_0_
Total	m-R	R	m

**Note:** V = number of Type I errors(false positive), T = number of type II errors(false negative).

**Table 2:** Parameter values for the transcription elements for the simulated dataset.

Basal Promoter Activity	Motif Effects^1^
10	*Negative Motifs*
50	−40
90	−80
130	*Positive Motifs*
163	20
180	40
200	60
250	800
300	120
500	150

1 **Note:** In addition to these two negative and six positive motifs, 42 motifs with no effects were included in the simulated dataset

**Table 3:** The Tests in Simulated Population and Permutated Population

Obs	tValue	DF	Motif	CWER	Exp	FWER	FDR adjusted p-value
1	12.61	331	2	<0.0001	0.0000	0.0000	0.0000
2	−12.07	352	7	<0.0001	0.0000	0.0000	0.0000
3	−17.63	341	8	<0.0001	0.0000	0.0000	0.0000
4	−7.68	325	6	<0.0001	0.0000	0.0000	0.0000
5	−5.73	353	5	<0.0001	0.0000	0.0000	0.0000
6	4.48	322	1	<0.0001	0.0005	0.0005	0.0001
7	−3.56	378	4	0.0004	0.0206	0.0204	0.0029
8	−2.97	325	3	0.0032	0.1600	0.1479	0.0200
9	−2.97	325	33	0.0032	0.1600	0.1479	0.0178
10	2.14	359	42	0.0328	1.6410	0.8062	0.1641
11	2.05	304	23	0.0417	2.0854	0.8758	0.1896
12	1.98	333	40	0.0484	2.4187	0.9110	0.2016
13	1.75	356	42	0.0817	4.0858	0.9832	0.3143
**Permutation Results**							
1	2.17	147	17	0.0320	1.5996	0.79802	1.5996
2	2.07	165	32	0.0401	2.0069	0.8656	1.0035
3	−1.76	144	34	0.0807	4.0367	0.98234	1.3456
4	−1.73	134	16	0.0853	4.2632	0.98592	1.0658
5	1.71	154	14	0.0892	4.4588	0.98842	0.8918
6	1.62	147	21	0.1071	5.3558	0.99528	0.8926
7	1.5	124	1	0.13669	6.8346	0.99892	0.9764

**Table 4:** The Functional Motif Candidates in the Fur-related Cluster

Type	tValue	CWER	exp	FWER	FDR adjusted p-	Motif Sequence
A	−10.53	0.0000	0.0000	0.0000	0.0000	GATAATAATTAT
A	−10.53	0.0000	0.0000	0.0000	0.0000	ATAATTATTATC
A	4.69	0.0001	0.2300	0.2064	0.0043	TAATGATTATC
B	−10.53	0.0000	0.0000	0.0000	0.0000	CGTAACGC
B	−5.58	0.0000	0.0000	0.0000	0.0017	CGTGACGC
B	−5.58	0.0000	0.0000	0.0000	0.0017	GCGTCACG
C	16.9	0.0000	0.0000	0.0000	0.0015	GCCGGA
C	16.9	0.0000	0.0000	0.0000	0.0015	TCCGGC

**Table 5:** type 3 tests of fixed effects

Effects	NDF	DDF	F value	Pr > F
Motif	3	262	9.99	<0.0001
Time*Motif	3	262	23.67	<0.0001
*Time*^2^	3	262	18.00	<0.0001

**Note:** NDF: numerator degrees of freedom; DDF: denominator degrees of freedom

**Table 6:** The estimations of main effects and interaction of the motif candidates

Effects	ML(s.e.)	REML(s.e.)	MIVQUE0(s.e.)
Motif A	0.3278(0.0854)	0.3278(0.0869)	0.3278(0.0869)
Motif B	−0.3569(0.0887)	−0.3569(0.0901)	−0.3569(0.0901)
Motif C	0.0438(0.1482)	0.0438(0.1507)	0.0438(0.1507)
Time * Motif A	0.0636(0.0436)	0.0636(0.0443)	0.0636(0.0443)
Time * Motif B	0.2965(0.0452)	0.2965(0.0460)	0.2965(0.0460)
Time* Motif C	0.6006(0.1129)	0.6006(0.1148)	0.6006(0.1148)
*Time*^2^ * Motif A	−0.0036(0.0047)	−0.0036(0.0048)	−0.0036(0.0048)
*Time*^2^ * Motif B	−0.0166(0.0049)	−0.0166(0.0050)	−0.0166(0.0050)
*Time*^2^ * Motif C	−0.1222(0.0185)	−0.1222(0.0188)	−0.1222(0.0188)

**Note:** ML: maximum likelihood method. REML: Restricted maximum likelihood method. MIVQUE0: minimum variance quadratic unbiased estimation method. S.E.: standard error.
